# From Farm to Fork: Antimicrobial-Resistant Bacterial Pathogens in Livestock Production and the Food Chain

**DOI:** 10.3390/vetsci12090862

**Published:** 2025-09-04

**Authors:** Ayman Elbehiry, Eman Marzouk

**Affiliations:** Department of Public Health, College of Applied Medical Sciences, Qassim University, P.O. Box 6666, Buraydah 51452, Saudi Arabia; e.marzouk@qu.edu.sa

**Keywords:** antimicrobial resistance (AMR), livestock production systems, zoonotic pathogens, One Health, public health, genomic surveillance, antimicrobial stewardship

## Abstract

Antimicrobial resistance (AMR) in livestock production systems has emerged as a major global health concern, threatening not only animal welfare and agricultural productivity but also food safety and public health. The widespread, and often poorly regulated, use of antimicrobials for growth promotion, prophylaxis, and metaphylaxis has accelerated the emergence and dissemination of resistant bacteria and resistance genes. These elements circulate across interconnected animal, environmental, and human ecosystems, driven by mobile genetic elements and amplified through the food production chain. It is estimated that more than two-thirds of medically important antimicrobials are used in animals, and AMR could cause millions of human deaths annually by mid-century if unchecked. In some livestock systems, multidrug-resistant *E. coli* prevalence already exceeds half of isolates, particularly in poultry and swine in low- and middle-income countries (LMICs). This narrative review provides a comprehensive overview of the molecular epidemiology, ecological drivers, and One Health implications of AMR in food-producing animals. We highlight key zoonotic and foodborne bacterial pathogens—including *Escherichia coli*, *Salmonella enterica*, and *Staphylococcus aureus*—as well as underappreciated reservoirs in commensal microbiota and livestock environments. Diagnostic platforms spanning phenotypic assays, PCR, MALDI-TOF MS, whole-genome sequencing, and CRISPR-based tools are examined for their roles in AMR detection, surveillance, and resistance gene characterization. We also evaluate current antimicrobial stewardship practices, global and regional surveillance initiatives, and policy frameworks, identifying critical implementation gaps, especially in low- and middle-income countries. Emerging sectors such as aquaculture and insect farming are considered for their potential role as future AMR hotspots. Finally, we outline future directions including real-time genomic surveillance, AI-assisted resistance prediction, and integrated One Health data platforms as essential innovations to combat AMR. Mitigating the threat of AMR in animal agriculture will require coordinated scientific, regulatory, and cross-sectoral responses to ensure the long-term efficacy of antimicrobial agents for both human and veterinary medicine.

## Simple Summary

Antimicrobial resistance (AMR) in livestock is a growing global threat that compromises animal health, food safety, and human medicine. Resistant bacteria such as *Escherichia coli* (*E. coli*), *Salmonella*, *Staphylococcus aureus (S. aureus*), and *Campylobacter* spread easily along the “farm-to-fork” chain, with commensals like *Enterococcus* and *Klebsiella* acting as hidden reservoirs of resistance genes. While modern tools such as PCR, MALDI-TOF MS, and genome sequencing are advancing AMR detection, major gaps remain in surveillance and regulation, especially in low- and middle-income countries (LMICs). Urgent One Health action—through antimicrobial stewardship, strict biosecurity, and innovative alternatives—is essential to contain this crisis and protect the future effectiveness of antibiotics.

## 1. Introduction

Animal-sourced food products—such as meat, milk, and eggs—are fundamental components of the global diet, providing high-quality proteins, essential fatty acids, and micronutrients that are often difficult to obtain from plant-based sources alone. These products are critical to nutritional security, especially among vulnerable populations [1]. As the global population grows and dietary patterns shift toward increased consumption of animal-based foods, livestock production systems have intensified to meet rising demand. While these intensified systems enhance productivity, they also create high-density environments that facilitate the emergence and spread of infectious diseases, including those caused by bacterial pathogens [2]. Of particular concern is the escalating development and transmission of antimicrobial-resistant (AMR) bacteria within these systems, largely driven by the widespread and frequently unregulated use of antimicrobials in animal agriculture [3].

Current estimates indicate that more than 70% of medically important antimicrobials are consumed in food-producing animals [4,5], and AMR could cause up to 10 million human deaths annually by 2050 if unchecked [6]. In intensive poultry production systems in Uganda, the prevalence of multidrug-resistant *E. coli* has been reported at approximately 62.7% [7]. This dynamic poses a significant threat not only to animal health and food safety but also to public health at large, reinforcing the need for integrated surveillance and stewardship strategies across the food production continuum.

Antimicrobials have been widely used in animal husbandry for decades, not only for the treatment of clinical infections but also for prophylactic and metaphylactic purposes, as well as historically for growth promotion [8]. While these practices have contributed to disease control and improved productivity, their extensive and often poorly regulated use has imposed significant selective pressure on microbial communities in livestock environments. This pressure has facilitated the emergence and proliferation of multidrug-resistant (MDR) bacterial strains, which pose a growing threat to both animal and human health. Current estimates indicate that approximately 70–73% of all antimicrobials deemed medically important for humans are consumed in food-producing animals, particularly in intensive farming systems [9,10]. Alarmingly, resistance genes encoding for tetracyclines, aminoglycosides, β-lactams, macrolides, and colistin have been increasingly detected in bacterial isolates from livestock, highlighting the critical role of primary production systems in the global antimicrobial resistance (AMR) crisis [11,12].

Among the most significant zoonotic and foodborne pathogens associated with AMR are *Escherichia coli* (*E. coli*), *Salmonella enterica* (*S. enterica*), *Campylobacter jejuni* (*C. jejuni*), *Listeria monocytogenes* (*L. monocytogenes*), *Staphylococcus aureus* (*S. aureus*), and *Enterococcus* spp. These organisms commonly inhabit the gastrointestinal tracts of food-producing animals and can persist in farm environments—contaminating animal housing, equipment, feed, and water. Through fecal shedding, these pathogens may enter the food chain and ultimately pose risks to human health via contaminated meat, milk, eggs, or direct contact with colonized animals [13]. Intensive livestock production systems, particularly those characterized by high animal density and antimicrobial use, serve as ecological niches that promote horizontal gene transfer among bacteria. This exchange is mediated by mobile genetic elements (MGEs) such as plasmids, integrons, transposons, and staphylococcal cassette chromosome mec (SCCmec) cassettes, contributing to the dissemination of multidrug resistance [14,15]. These AMR pathogens can reach consumers through the “farm-to-fork” continuum, including via undercooked or raw animal-derived foods, cross-contamination during food preparation, or exposure to contaminated surfaces [16]. This complex web of transmission underscores the need for a One Health approach to AMR mitigation, addressing microbial hazards at the animal, human, and environmental interface.

While this review highlights major zoonotic and foodborne pathogens such as *E. coli*, *Salmonella*, *Campylobacter*, *Listeria*, *S. aureus*, and *Enterococcus* spp., we acknowledge that other emerging pathogens—including *Acinetobacter baumannii, Pseudomonas aeruginosa*, and *Clostridioides difficile*—also contribute significantly to the global AMR burden. These species are recognized in international priority pathogen lists [17] and global burden analyses [18], but were not covered in detail here to maintain focus on the most widely reported and epidemiologically relevant bacteria in livestock and food systems.

This growing threat underscores the essential need for a holistic One Health approach to AMR, one that bridges human, animal, and environmental health through integrated surveillance, prevention, and control strategies. The World Health Organization (WHO) [19] highlights how the Quadripartite bodies—comprising the WHO, Food and Agriculture Organization of the United Nations (FAO), World Organization for Animal Health (WOAH), and United Nations Environment Programme (UNEP)—formalized this integration in the One Health Joint Plan of Action (2022–2026), which outlines strategic frameworks for capacity strengthening, multisectoral coordination, and collaborative governance across sectors. However, several studies report persistent gaps in implementing these frameworks, particularly in low- and middle-income countries (LMICs). Cella et al. [20] and Delpy et al. [21] document how LMICs often contend with fragmented AMR surveillance systems, weak laboratory infrastructure, and inconsistent policy enforcement—hindering effective One Health response efforts

While several high-income countries have implemented robust antimicrobial stewardship (AMS) programs in the agricultural sector, global progress remains inconsistent. Within the European Union, the Farm to Fork strategy aims to reduce sales of veterinary antimicrobials by 50% by 2030, a target that is increasingly attainable given the observed 28% reduction in antimicrobial usage in food-producing animals and aquaculture between 2018 and 2022 [22]. Denmark’s Yellow Card initiative, introduced in 2010, has demonstrated notable success in curbing antibiotic consumption in pig production by imposing usage thresholds and sanctioning high-use farms, leading to substantial and sustained reductions in antimicrobial administration [23]. In contrast, many LMICs, particularly in parts of Asia, Latin America, and sub-Saharan Africa, continue to report high levels of antibiotic use in livestock, often in the absence of stringent regulatory oversight. This unmonitored use contributes significantly to the emergence of antimicrobial-resistant bacteria and facilitates their transmission along the food chain, elevating the risk of zoonotic spillover and undermining global AMR containment efforts [24,25].

In parallel, technological advancements have greatly improved the detection and characterization of AMR. Traditional phenotypic assays are now routinely complemented by advanced molecular diagnostics—including polymerase chain reaction (PCR), loop-mediated isothermal amplification (LAMP), matrix-assisted laser desorption/ionization time-of-flight mass spectrometry (MALDI-TOF MS), whole-genome sequencing (WGS), and metagenomic next-generation sequencing (mNGS)—all of which provide superior sensitivity and specificity for detecting both established and emerging resistance determinants. PCR remains a rapid and reliable method for the identification of known resistance genes, whereas LAMP is particularly valuable in low-resource settings due to its high sensitivity and tolerance to inhibitors. MALDI-TOF MS has transformed microbial diagnostics by enabling fast, accurate species identification coupled with emerging applications in AMR profiling [26]. WGS offers comprehensive pathogen characterization and robust prediction of resistance phenotypes, often showing strong concordance with conventional susceptibility testing [27]. mNGS further extends these capabilities by allowing unbiased detection of resistance genes directly from complex clinical, food, or environmental samples without the need for prior culture [28]. Despite their promise, the widespread adoption of these technologies in resource-limited regions remains constrained by high costs, technical complexity, and infrastructure requirements [29].

Concurrently, a range of innovative mitigation strategies is gaining traction. Bacteriophage therapy shows promise in treating animal infections like bovine mastitis and reducing pre-harvest contamination [30], while alternatives such as antimicrobial peptides, probiotics, essential oils, nanomaterials, CRISPR-based antimicrobials, and AI-driven prediction models are emerging as viable tools to prevent and manage AMR threats [31,32]. Despite encouraging results from in vitro and pilot trials, many of these novel interventions face significant barriers to large-scale deployment, including regulatory challenges, standardization issues, and integration into conventional animal health systems [31].

This review aims to provide a comprehensive overview of AMR in livestock production systems within a One Health framework. We synthesize current knowledge on the major bacterial pathogens of concern, the drivers of resistance emergence, and the transmission routes that connect farm environments to human populations through food, direct contact, and the wider ecosystem. Particular attention is given to surveillance gaps, diagnostic innovations, and policy challenges in low- and middle-income countries, where regulatory oversight remains limited. In addition, *S. aureus* is highlighted as a case study to illustrate farm-to-fork transmission pathways in greater detail, while other major pathogens are addressed in broader sections.

By adopting this integrated perspective, the review underscores the need for coordinated, species-tailored, and cross-sectoral strategies to contain AMR and protect both animal and human health. In low-resource systems, short-term and feasible measures include restricting the use of critically important antimicrobials (CIAs) through basic regulatory guidance, introducing low-cost biosecurity improvements such as hygiene training, vaccination, and improved farm sanitation, and strengthening surveillance capacity using affordable diagnostic tools (e.g., phenotypic AST, portable qPCR, and LAMP assays). These interventions represent scalable and immediately actionable steps, while more resource-intensive strategies—such as WGS-based surveillance or comprehensive stewardship frameworks—remain longer-term goals [33].

This review adopts a narrative rather than systematic (e.g., PRISMA) approach, as our objective was to integrate diverse sources—including epidemiological studies, surveillance data, and policy reports from FAO, WHO, WOAH, and EFSA—that extend beyond the scope of systematic frameworks. While this format enhances breadth and interpretative depth, we acknowledge its limitations in transparency and reproducibility compared to systematic methodologies. For this review, the relevant literature was retrieved primarily from PubMed, Scopus, and Web of Science, covering the period 2000–2025, and restricted to English-language publications. Key international reports and policy documents from FAO, WHO, WOAH, and EFSA were also included to capture essential surveillance and governance frameworks.

**Figure 1:**
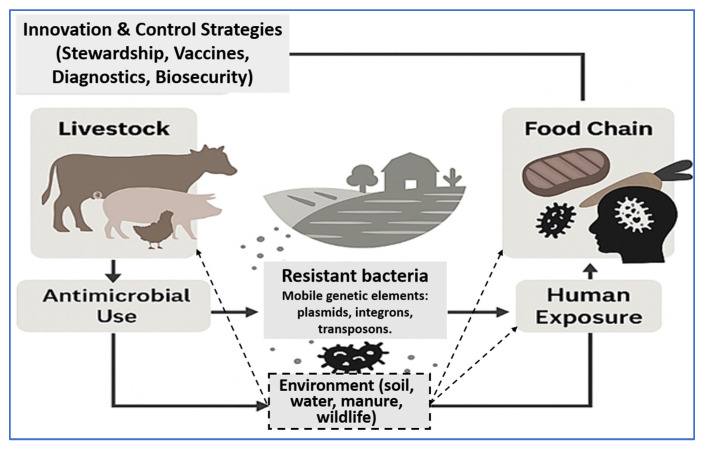
Farm-to-fork AMR transmission dynamics and control feedback loop. Antimicrobial use in livestock promotes resistant bacteria that spread through the food chain, direct human contact, and environmental reservoirs (soil, water, manure, wildlife). Mobile genetic elements (plasmids, integrons, transposons) amplify resistance transfer across compartments. Innovation and control strategies—such as antimicrobial stewardship, vaccination, diagnostics, and biosecurity—intervene at multiple points to mitigate AMR dissemination under a One Health framework.

Figure 1 illustrates the farm-to-fork transmission cycle of AMR in livestock production systems. Antimicrobial use in animals leads to the development of resistant bacteria, which spread to humans through the food chain, direct exposure, and environmental reservoirs such as soil, water, manure, and wildlife. Mobile genetic elements further facilitate the dissemination of resistance across these compartments. Innovation and control strategies—including stewardship, vaccination, diagnostics, and biosecurity—provide intervention points to disrupt this cycle within a One Health framework.

## 2. Major AMR Bacterial Pathogens in Livestock

The intensification of livestock farming, driven by global demand for animal-sourced food, has created ideal conditions for the emergence, amplification, and dissemination of AMR bacteria. High animal densities, suboptimal biosecurity, and the widespread use of antimicrobials for disease prevention, metaphylaxis, and growth promotion contribute to sustained selective pressure favoring resistant strains [34]. Numerous studies have demonstrated that such environments support the proliferation of diverse antimicrobial resistance genes (ARGs), often located on MGEs such as plasmids, integrons, and transposons, thereby facilitating horizontal gene transfer between commensal and pathogenic bacteria [35]. These resistant organisms are capable of colonizing livestock gastrointestinal and mucosal surfaces, where they may persist asymptomatically and be shed into the environment through feces, urine, or respiratory secretions. From there, AMR bacteria can reach humans via multiple transmission routes, including direct animal contact, the consumption of contaminated meat, milk, or eggs, and through environmental pathways such as manure runoff, irrigation water, and aerosols [36,37].

A growing body of genomic surveillance data has confirmed the existence of clonal overlap and gene-sharing events between livestock-derived and human clinical isolates, underscoring the zoonotic and foodborne potential of these resistant strains [38,39]. This interconnectedness reinforces the necessity of adopting a One Health approach, integrating surveillance and control measures across human, animal, and environmental sectors. Such strategies include the implementation of AMS programs in animal husbandry, improvement of farm hygiene and vaccination practices, control of waste and effluent discharge, and restriction of critically important antimicrobials (CIAs) in food-producing animals. Understanding the key AMR pathogens in livestock and mapping their resistance profiles across species and production systems are foundational to guiding evidence-based interventions aimed at mitigating AMR threats globally.

### 2.1. E. coli and Salmonella enterica: High-Risk Zoonotic Enterobacteriaceae

Among zoonotic foodborne pathogens, *E. coli* represents a critical public health concern due to its widespread prevalence in poultry, cattle, and swine. Both Shiga toxin-producing *E. coli* (STEC) and extraintestinal pathogenic strains (ExPEC) are frequently associated with multidrug resistance, particularly through the acquisition of extended-spectrum β-lactamase (ESBL) genes such as *bla_CTX-M* and *bla_SHV*. In addition, the emergence of plasmid-mediated colistin resistance via the *mcr-1* gene has significantly complicated treatment options. The *mcr-1* gene was first identified in the *E. coli* strain SHP45, isolated from pigs in China in 2015, and has since been reported in animal, human, and environmental samples across all continents, reflecting its rapid and global dissemination within food production systems [40,41].

Similarly, *Salmonella enterica*, a leading etiological agent of foodborne infections worldwide, has exhibited increasing resistance to CIAs, particularly β-lactams and fluoroquinolones. Resistance is commonly mediated by genes such as *bla_TEM*, *bla_CTX-M*, and *qnrB*, often located on integrons and plasmids that enhance their horizontal transfer potential. Recent surveillance studies in poultry production systems have identified a high prevalence of class 1 integrons and ESBL-encoding genes among *Salmonella Enteritidis* and *Salmonella typhimurium* isolates, underscoring the potential for rapid dissemination within and between farms [42,43]. The convergence of virulence and resistance traits in these pathogens highlights their significant zoonotic and foodborne transmission risk, necessitating urgent and integrated control strategies.

We selected *bla*CTX-M, *mcr-1*, and *tetM* as representative resistance determinants because of their widespread occurrence in livestock and their recognized importance for both veterinary and human health. *bla*CTX-M variants drive much of the extended-spectrum β-lactamase resistance observed in *E. coli*, *mcr-1* marked the first discovery of a plasmid-mediated colistin resistance gene of global concern, and *tetM* exemplifies transferable tetracycline resistance across a broad range of Gram-positive and Gram-negative bacteria. To place these mechanisms in context, Table 1 summarizes their prevalence across continents, underscoring their global epidemiological significance in livestock production. Collectively, these genes illustrate how a few resistance determinants can serve as powerful markers of AMR dissemination worldwide. The following sections build on this foundation by examining additional foodborne pathogens, including *C. jejuni* and *L. monocytogenes*, which highlight the further diversity and complexity of resistance mechanisms along the farm-to-fork continuum.

### 2.2. Campylobacter jejuni and Listeria monocytogenes: AMR in Foodborne Pathogens

*Campylobacter jejuni* (*C. jejuni*), the leading bacterial cause of human gastroenteritis globally, is predominantly associated with poultry, particularly under intensive production systems. The emergence of AMR in *C. jejuni* has become a major public health concern, especially resistance to fluoroquinolones and macrolides—two of the most critical drug classes for human treatment. Fluoroquinolone resistance is primarily conferred by point mutations in the *gyrA* gene, most commonly a C257T substitution that results in a Thr86Ile amino acid change, while macrolide resistance is associated with mutations in the *23S rRNA* gene and the overexpression of the cmeABC efflux pump system, which contributes to multidrug resistance phenotypes [50,51]. Additionally, tet (O), a ribosomal protection protein gene, has been widely detected in Campylobacter isolates from broiler chickens, with reported prevalence rates exceeding 70% in some regions, including Latin America and Asia [52]. Although *Listeria monocytogenes* remains intrinsically susceptible to many antibiotics, emerging resistance to tetracyclines and macrolides has been reported in foodborne and animal-derived isolates. Resistance genes such as *tetM*, *tetS*, and *ermB* have been detected in isolates from dairy cattle, goats, and contaminated milk and cheese products. These genes are frequently located on conjugative transposons such as Tn916, facilitating horizontal gene transfer between Listeria and other Gram-positive bacteria in shared environments [53,54,55]. The growing documentation of such resistance determinants in zoonotic and foodborne pathogens highlights the need for continuous surveillance and prudent AMU in food animal production systems.

### 2.3. S. aureus: The Rise in Livestock-Associated MRSA

*S. aureus*, particularly livestock-associated methicillin-resistant strains (LA-MRSA), poses a growing public health threat within intensive animal production systems [56]. Among these, the clonal lineage ST398, belonging to clonal complex CC398, is the most widely reported and has been extensively documented in pigs, cattle, and, increasingly, poultry [57,58]. This lineage is commonly associated with the carriage of the *mecA* gene, which encodes penicillin-binding protein 2a (PBP2a) and confers resistance to β-lactam antibiotics, including methicillin. In addition, ST398 isolates frequently harbor the *tetM* gene, mediating resistance to tetracyclines, and the *ermC* gene, conferring macrolide resistance—reflecting the selective pressure from routine antibiotic use in livestock production [59,60]. The zoonotic potential of ST398 is well-established. Numerous studies employing WGS and spa typing have demonstrated that isolates from farm workers are often genetically indistinguishable from those found in animals, supporting direct transmission between livestock and humans [61,62]. For example, matched *S. aureus* ST398 isolates from pigs and swine workers in European and Southeast Asian countries shared identical spa types, resistance genes, and SCCmec elements [63]. Epidemiological surveillance in Denmark and the Netherlands has also shown that human infections caused by ST398—including skin, wound, and bloodstream infections—have increased in parallel with LA-MRSA prevalence in pig farms, raising concerns over its infiltration into the broader community [64]. The emergence of LA-MRSA in livestock environments thus presents a dual challenge; it threatens animal health and productivity, while simultaneously serving as a reservoir of antimicrobial-resistant pathogens with the potential to cause severe infections in humans, particularly those with occupational exposure. This underscores the importance of integrated One Health surveillance and AMS across both veterinary and public health sectors.

### 2.4. Opportunistic and Commensal Reservoirs: Enterococcus spp. and K. pneumoniae

Beyond primary zoonotic pathogens, several opportunistic and commensal bacteria in livestock contribute significantly to the AMR burden. *Enterococcus faecalis* (*E. faecalis*) and *Enterococcus faecium* (*E. faecium*), common inhabitants of the gastrointestinal tracts of poultry and swine, are particularly adept at acquiring and disseminating resistance genes. Of greatest concern is their ability to develop high-level resistance to vancomycin through the acquisition of *vanA* and *vanB* operons, as well as to aminoglycosides via the bifunctional enzyme encoded by *aac(6′)-Ie-aph(2″)-Ia* [65]. These resistance genes are frequently located on transferable elements such as transposons (e.g., Tn5281), facilitating horizontal gene transfer to other Gram-positive bacteria, including *S. aureus* and *L. monocytogenes* [66]. This capacity positions enterococci as important reservoirs and vectors of clinically significant resistance traits across the animal–human interface. *Klebsiella pneumoniae* (*K. pneumoniae*), historically associated with mastitis in dairy cattle and respiratory infections in poultry, is also emerging as a major MDR pathogen in animal production environments. Animal-derived isolates have increasingly been found to carry carbapenemase-encoding genes such as *bla_KPC* and *bla_OXA-48*, as well as the mobile colistin resistance gene *mcr-1*—previously thought to be limited to human clinical isolates [67,68].

Recent genomic surveillance and meta-analyses have identified carbapenemase (*bla_KPC*, *bla_OXA-48*-like) and colistin resistance (*mcr-1*) genes in *K. pneumoniae* isolates from livestock in Europe, Asia, and South America, underscoring the global distribution and zoonotic potential of these pathogens [69,70]. These findings underscore the risk posed by MDR *K. pneumoniae* in animal reservoirs, particularly in contributing to the environmental dissemination of resistance determinants and their potential to seed nosocomial infections in humans. The collective evidence reinforces the importance of including commensal and opportunistic bacteria in AMR monitoring and One Health surveillance frameworks.

### 2.5. S. pseudintermedius: An Emerging Zoonotic Threat

In addition to its veterinary relevance, *Staphylococcus pseudintermedius* (*S. pseudintermedius*), a coagulase-positive staphylococcus primarily associated with dogs, has emerged as a zoonotic pathogen of increasing concern, particularly in the context of AMR. Methicillin-resistant *S. pseudintermedius* (MRSP) strains commonly harbor the *mecA* gene, which confers resistance to β-lactam antibiotics, and the *aadD* gene, which mediates aminoglycoside resistance [71]. Although historically restricted to companion animals, MRSP has been increasingly reported in ruminants and livestock farm environments, suggesting a broader ecological presence and adaptation [72,73]. The zoonotic potential of MRSP is now well established. Genomic and epidemiological studies have confirmed the transmission of MRSP from dogs to humans, particularly among pet owners and veterinary professionals. Comparative genome sequencing has revealed near-identical MRSP strains in canine and human hosts, often sharing the same sequence types (ST71 and ST45), SCCmec elements, and resistance gene profiles [72]. In some cases, human MRSP infections have resulted in skin and soft tissue infections or bacteremia, particularly in individuals with close and prolonged contact with colonized animals [74]. Moreover, MRSP strains isolated from livestock workers and farm animals have demonstrated multidrug resistance to β-lactams, tetracyclines, macrolides, and fluoroquinolones, raising concerns about occupational exposure and the pathogen’s capacity to spread beyond the veterinary setting [75]. Given its increasing clinical relevance and capacity for interspecies transmission, *S. pseudintermedius* should no longer be viewed solely as a veterinary pathogen but rather as an emerging zoonotic agent with significant implications for One Health AMR surveillance and infection control efforts.

### 2.6. Host-Specific Dynamics and Transmission Pathways

Host species, antimicrobial usage patterns, and farming practices play pivotal roles in shaping the distribution and dynamics of AMR bacteria within livestock production systems. In poultry, the high-density rearing environment, combined with routine administration of antibiotics, fosters reservoirs of ESBL-producing *E. coli* and fluoroquinolone-resistant *C. jejuni* [43,76]. Longitudinal studies conducted on broiler farms have consistently identified ESBL-positive *E. coli* in both avian hosts and their surrounding environments, indicating persistent and widespread contamination [77]. Similarly, fluoroquinolone-resistant *Campylobacter* strains, often carrying *gyrA* mutations and *cmeABC* efflux systems, are highly prevalent in poultry carcasses and processing facilities [78]. In swine production, the frequent use of tetracyclines, macrolides, and β-lactams has been linked to the emergence of LA-MRSA, particularly the clonal lineage ST398, and the dissemination of vancomycin-resistant *E. faecium* (VRE). Environmental and animal sampling in commercial pig farms has demonstrated the concurrent presence of LA-MRSA and VRE, with evidence of horizontal transmission between animals, farmworkers, and surrounding habitats [78].

In contrast, dairy cattle are frequently associated with antimicrobial-resistant strains of *L. monocytogenes*, *K. pneumoniae*, and *S. aureus*, particularly in cases of bovine mastitis. Recent meta-analyses have reported increasing rates of multidrug-resistant *K. pneumoniae* in mastitic milk samples, often harboring *bla_CTX-M* and *mcr-1* genes [79]. Similarly, resistant *S. aureus* and *Streptococcus uberis* strains from dairy herds have been shown to exhibit resistance to β-lactams, macrolides, and lincosamides, complicating treatment protocols and increasing the risk of persistent infections [80,81]. Importantly, the presence of MGEs—such as plasmids, transposons, and class 1 integrons—across these production systems facilitates the horizontal transfer of AMR genes among bacterial populations. For instance, plasmid-mediated ESBL and colistin resistance genes have been detected in hatcheries and neonatal poultry, underscoring the potential for early-life AMR transmission and amplification within the food chain [82]. These complex, host-specific resistance patterns highlight the necessity for integrated, species-tailored surveillance and intervention strategies to effectively mitigate the propagation of AMR from farm to fork.

Table 2 provides a concise overview of the major AMR bacterial species identified in livestock, categorized by host animal, key resistance genes, associated clinical manifestations, and potential transmission routes to humans. The table highlights both zoonotic pathogens—such as *Escherichia coli*, *Salmonella enterica*, and *Staphylococcus aureus*—and opportunistic or commensal bacteria including *E. faecium*, *K. pneumoniae*, and *S. pseudintermedius*. It underscores the diversity of resistance determinants, such as *bla_CTX-M*, *mecA*, *vanA*, and *mcr-1*, which are frequently located on MGEs and facilitate gene exchange across bacterial populations and host species. This summary supports the One Health perspective by linking livestock-associated AMR to public health risks through direct animal contact, food consumption, and environmental exposure pathways.

## 3. Drivers of AMR in Primary Animal Production

The emergence and persistence of AMR in livestock production is not driven by a single factor, but rather by a complex interplay of biological, environmental, and anthropogenic forces that together create a fertile environment for the selection, amplification, and dissemination of resistant bacteria. These drivers act synergistically within intensive animal production systems, directly and indirectly shaping microbial ecology and resistance gene dynamics [94]. Key contributors include the widespread misuse of antimicrobials, poor biosecurity, ineffective waste management, and co-selection from heavy metals and disinfectants—all of which promote resistance development and facilitate horizontal gene transfer [95]. For example, excessive supplementation with zinc and copper in pig farming has been strongly associated with the persistence of plasmid-borne resistance genes, even in the absence of antibiotic pressure [96]. Moreover, intensified livestock operations have been linked to increased loads of resistance genes in feces, wastewater, and adjacent soil and crops, highlighting the broader ecological spillover of AMR traits [34]. This underscores the critical need for coordinated One Health strategies that acknowledge and address the interconnected nature of animal husbandry, environmental contamination, and human health risks [37].

### 3.1. Non-Therapeutic Use of Antimicrobials: Growth Promotion, Prophylaxis, and Metaphylaxis

One of the most pressing and well-documented contributors to AMR in animal agriculture is the non-therapeutic use of antimicrobials for growth promotion, disease prevention, and metaphylaxis. In many LMICs, antibiotics are routinely administered via feed or water, often without veterinary oversight or proper dosing protocols [97]. While intended to enhance productivity and prevent disease, these practices exert chronic selective pressure on animal microbiota, fostering the emergence and persistence of MDR organisms in clinically healthy animals [98,99]. The continued use of CIAs for human medicine—such as colistin, β-lactams, macrolides, and tetracyclines—in food-producing animals has led to the alarming spread of resistance determinants including *mcr-1*, *bla_CTX-M*, and *ermB*. These genes, frequently carried on mobile plasmids, have been recovered not only from animal isolates but also from environmental and human samples, illustrating their rapid and far-reaching dissemination [40,41,100]. These trends demand urgent global stewardship reforms, emphasizing the phase-out of non-essential AMU and the enforcement of regulatory policies to protect public health.

### 3.2. Environmental Stressors and Farm Management Practices

Environmental stressors and suboptimal management practices in intensive farming systems play a pivotal role in AMR propagation. Factors such as overcrowding, poor ventilation, inadequate sanitation, and weak biosecurity measures increase the microbial load in animal environments and induce immunosuppression, necessitating prophylactic AMU and further fueling resistance selection [101,102]. A major pathway for environmental AMR transmission is through the mismanagement of livestock waste. Manure, slurry, and bedding are often laden with antibiotic residues, resistant bacteria, and resistance genes. When applied to fields as untreated organic fertilizer, these materials enrich the soil resistome and promote horizontal gene transfer among soil and plant-associated microbes [103,104]. Even in the absence of ongoing antibiotic exposure, residual antibiotics in manure-amended soils continue to exert selective pressure, favoring the persistence of resistance determinants [105].

The risk is further amplified by the leaching of these contaminants into surface water, groundwater, and irrigation systems, facilitating the transport of ARGs into broader environmental and human domains. Surveillance studies across multiple continents have documented this “resistance spillover”, linking agricultural runoff to the enrichment of ARGs in aquatic ecosystems [106]. The frequent detection of MGEs such as plasmids and integrons in manure-treated soils further emphasizes the ecological connectivity of AMR spread [107]. Implementing advanced waste treatment systems—including composting, anaerobic digestion, and constructed wetlands—along with improved farm hygiene and housing, represents a critical intervention to mitigate environmental AMR emissions under a One Health paradigm.

### 3.3. Horizontal Gene Transfer via Mobile Genetic Elements

Horizontal gene transfer (HGT) mediated by MGEs constitutes a cornerstone mechanism of AMR evolution in livestock environments. MGEs such as plasmids, integrons, transposons, insertion sequences, and bacteriophages enable rapid acquisition and dissemination of resistance genes across phylogenetically diverse bacterial species and ecological niches [108]. Plasmid-encoded genes like *bla_CTX-M*, *mcr-1*, *qnrS*, and *vanA* are frequently identified in resistant isolates of *E. coli*, *K. pneumoniae*, and *E. faecium* from pigs, poultry, and cattle. These plasmids often carry additional accessory genes that enhance bacterial survival under stress conditions, increasing their fitness and transmissibility [109,110]. Class 1 integrons, composite transposons such as Tn21 and Tn1546, and insertion elements like IS26 further promote the assembly and mobilization of multiple resistance traits [111,112]. Livestock environments harbor numerous “hotspots” for gene exchange—including biofilms in water troughs, manure lagoons, shared bedding, and processing equipment—where close microbial contact facilitates efficient HGT. These findings highlight the urgent need to integrate MGE surveillance, upgrade biosecurity, and prioritize waste decontamination in AMR control programs grounded in the One Health framework [113].

### 3.4. Co-Selection Pressures from Heavy Metals and Disinfectants

AMR selection in animal agriculture is not solely driven by antibiotic use. Co-selection pressures from heavy metals (e.g., copper, zinc) and disinfectants—especially quaternary ammonium compounds—have emerged as significant contributors to resistance evolution. These agents, widely used as feed additives, supplements, and hygiene agents, exert unintended selective pressure on microbial communities [114,115]. Resistance genes to heavy metals and biocides are frequently co-located with antibiotic resistance genes on the same plasmids or integrons, enabling bacteria exposed to one compound to simultaneously gain resistance to multiple antimicrobial classes [116]. For instance, plasmids co-harboring *mcr-1, tet(M)*, and heavy metal resistance genes have been detected in *E. coli* isolates from swine, revealing the potential of metal exposure to drive multidrug resistance [117].

Long-term heavy metal supplementation in pig and poultry diets has been associated with elevated levels of *bla_CTX-M*, *sul1*, and *mcr-1* in feces, manures, and surrounding soils [118,119]. Similarly, QAC resistance genes such as *qacEΔ1* often co-occur with class 1 integrons, exacerbating the horizontal spread of resistance [120]. Notably, metagenomic data suggest that heavy metal concentrations in livestock waste may better predict ARG abundance than antibiotic residues themselves. These insights call for comprehensive AMR containment policies that extend beyond antibiotics to include heavy metals, disinfectants, and broader chemical management under the One Health lens.

### 3.5. Summary and One Health Implications

**Figure 2:**
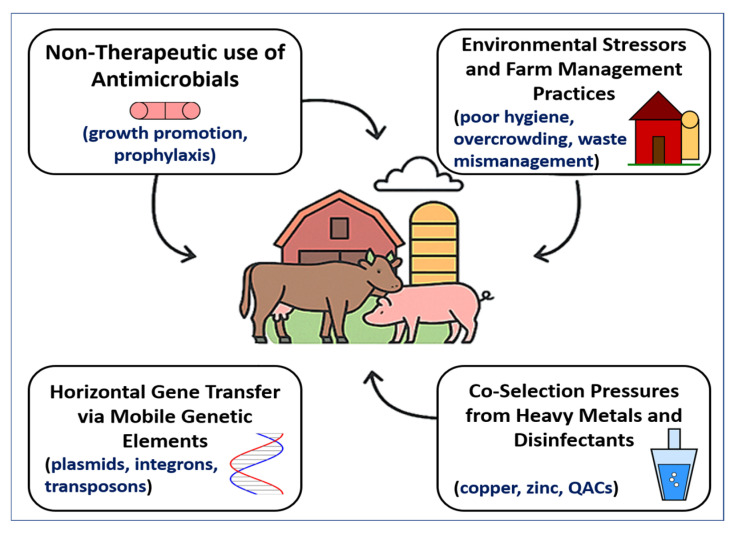
Drivers of AMR in primary animal production. A conceptual diagram showing four interconnected drivers of AMR in livestock systems: (1) non-therapeutic antimicrobial use (growth promotion, prophylaxis); (2) environmental stressors and poor farm management (hygiene, overcrowding, waste mismanagement); (3) horizontal gene transfer via mobile genetic elements (plasmids, integrons, transposons); and (4) co-selection pressures from heavy metals and disinfectants (copper, zinc, quaternary ammonium compounds). These drivers act synergistically to promote the selection and dissemination of resistant bacteria within intensive farming systems and across the animal–environment–human interface.

The convergence of antimicrobial overuse, environmental contamination, mobile genetic exchange, and chemical co-selection fosters a vicious and self-sustaining cycle of AMR in primary animal production. This cycle is perpetuated by high-density farming, routine antimicrobial inputs, and lax waste control, which together enable resistant microbes to flourish and spread across animal, environmental, and human domains. As depicted in Figure 2, these interconnected drivers collectively shape the emergence and dissemination of AMR. The downstream consequences include contamination of agricultural fields with resistance-laden manure, runoff into water bodies, and the infiltration of resistance genes into the food chain, soil, crops, and even wildlife [121]. The threat is compounded by the diminishing efficacy of last-resort antimicrobials critical for human medicine, thereby escalating the clinical burden of difficult-to-treat infections [122,123].

To effectively interrupt this cycle, a holistic, multisectoral response is imperative. Core priorities include (1) restricting the use of critically important antibiotics in livestock [99]; (2) improving on-farm hygiene, ventilation, and manure processing systems [121]; (3) regulating heavy metal feed additives to reduce co-selection pressure; (4) enhancing integrated AMR surveillance across sectors [124]; and (5) promoting non-antibiotic alternatives such as vaccination, probiotics, bacteriophages, and precision farming tools [125]. Ultimately, addressing AMR in animal production is not solely a veterinary concern but a global public health imperative. Success will hinge on coordinated One Health strategies that align scientific innovation with policy enforcement, industry engagement, and societal accountability.

### 3.6. Insects as Emerging AMR Reservoirs

Insects are increasingly promoted as a sustainable alternative to conventional livestock, with applications ranging from animal feed in poultry, aquaculture, and swine to direct human consumption. Their high protein content, rapid growth, efficient feed conversion, and reduced environmental footprint have accelerated the global expansion of insect farming [126,127]. This sector is recognized for its contribution to food and feed security while mitigating environmental pressures of traditional animal agriculture [128]. However, insect farming is not exempt from the One Health challenge of AMR. Rearing environments, substrates, and microbiota can harbor resistance genes, raising concerns that insects may serve as overlooked reservoirs of AMR.

Studies have reported ARGs in farmed insects. De Smet et al. [129] detected tetracycline- and sulfonamide-resistance genes in black soldier fly (*Hermetia illucens*) larvae, likely linked to antimicrobial residues in substrates. Garofalo et al. [130] identified transferable genes such as *tet(M)* and *erm(B)* in the gut microbiota of mealworms (*Tenebrio molitor*) and crickets (*Acheta domesticus*). These findings suggest that organic waste streams or manures enriched with resistant bacteria may promote horizontal gene transfer within insect guts, creating new ecological niches for AMR dissemination.

Processed insect meals, now approved for aquaculture and poultry feeds in Europe, may carry ARGs that persist through feed processing and interact with animal gut ecosystems. Osimani et al. [131] warned that insects could act as amplifiers of resistance determinants in integrated food systems. High-density farming conditions—crowded larvae and frequent substrate replenishment—further mirror intensive livestock practices that accelerate microbial exchange.

Nevertheless, evidence indicates that farmed insects harbor fewer clinically relevant ARGs than conventional livestock, reflecting their limited exposure to antimicrobials and simpler gut microbiota [129,131]. Comparative studies hypothesize a reduced abundance and dissemination potential of ARGs in insects relative to livestock [132]. Yet, rapid industrial growth and reliance on waste-based substrates raise concerns, as these can contain resistant bacteria and antimicrobial residues conducive to horizontal transfer [126,133]. Without surveillance, insects could act as “silent vectors”, facilitating ARG movement into livestock systems or human microbiota [134].

From a One Health perspective, integrating insect farming into AMR monitoring frameworks is essential. Mapping the insect resistome, tracking gene persistence through processing, and evaluating transfer risks should be prioritized. Reinforcing substrate regulations—particularly for manure and food waste—is critical to reduce ARG introduction [126,133]. Comparative analyses with conventional feed systems will clarify risks. In sum, while insects offer sustainability benefits, their role in AMR ecology remains underexplored. Recognizing insects as potential resistance reservoirs and embedding them in One Health surveillance will safeguard their contribution to sustainable protein production [134].

## 4. Farm-to-Fork Transmission Pathways of AMR *Staphylococcus* spp.

The dissemination of antimicrobial-resistant Staphylococcus species from farm environments to humans follows a multidimensional farm-to-fork continuum that includes fecal shedding, biofilm formation, slaughterhouse contamination, and household exposure. These interconnected contamination nodes enable the persistence and selection of resistant bacteria and mobile genetic elements. Understanding these transmission pathways is essential for designing targeted interventions to break zoonotic AMR cycles and protect public health [56,135].

### 4.1. Pre-Harvest Contamination: Fecal Shedding, Biofilms, and Milking Environments

Pre-harvest contamination represents a critical entry point for antimicrobial-resistant *Staphylococcus* spp. into the food chain. Colonized livestock—including dairy cows, goats, and sheep—shed *S. aureus* and coagulase-negative staphylococci (CoNS) through feces, nasal secretions, skin lesions, and infected udders. These bacteria readily contaminate farm environments, including bedding, feeding equipment, and milking facilities, where they can persist for extended periods [136]. Milking clusters, pipelines, and teat cups are particularly vulnerable to colonization, often harboring resistant staphylococcal biofilms that significantly hinder sanitation efforts and serve as reservoirs for recurrent contamination [137,138,139]. Biofilm formation enhances bacterial resistance not only to antibiotics but also to disinfectants and environmental stressors, promoting the persistence and reintroduction of MDR strains during subsequent milking cycles.

Molecular surveillance studies have consistently identified resistance determinants such as *mecA*, *tet(K)*, *tet(M)*, *erm(A/B/C)*, and *blaZ* within *S. aureus* isolates from raw milk samples in both smallholder and commercial dairy operations [140,141]. These findings underscore the importance of stringent pre-harvest hygiene protocols, particularly during milking, to minimize the introduction of resistant bacteria into bulk milk supplies. Furthermore, close contact between farm workers and colonized animals facilitates the bidirectional transmission of staphylococci, with humans serving as both recipients and reservoirs.

Occupational exposure has been associated with transient or persistent nasal and dermal carriage of livestock-associated *S. aureus* and CoNS, some of which harbor transferable resistance genes, contributing to the reverse zoonotic amplification of AMR [142,143]. This highlights the need for personal protective measures, regular screening, and education of farm personnel as part of integrated AMR control strategies. Together, these dynamics at the pre-harvest stage lay the foundation for downstream contamination and human exposure, reinforcing the necessity of One Health–aligned interventions that target the farm environment as the first critical control point in the AMR transmission chain.

### 4.2. Harvest and Post-Harvest Contamination: Slaughter, Processing, and Packaging

The harvest and post-harvest phases of livestock production represent critical control points for the introduction and dissemination of AMR *Staphylococcus* spp. During slaughter and carcass processing, resistant strains can be transferred from colonized skin, nasal passages, or mucosal tissues to meat surfaces, particularly under conditions of inadequate hygiene or improper handling. Environmental surfaces, such as scalding tanks, cutting boards, conveyor belts, and workers’ gloves, serve as major vectors for cross-contamination when sanitation protocols are not rigorously enforced [144]. Multiple studies have identified methicillin-resistant *S. aureus* (MRSA) and MDR *S. aureus* in meat products including poultry, beef, pork, and processed items such as sausages. These isolates often harbor a broad array of resistance determinants, including *mecA*, *tet(M)*, *erm(B)*, and various SCCmec elements [145,146,147]. Genotypic profiling has revealed the frequent presence of SCCmec types IV and V, which are associated with community- and livestock-associated MRSA clones. These resistance elements often coexist with virulence factors, compounding their public health threat.

Recent meta-analyses have reinforced the significance of this issue. A global systematic review by Xing et al. [148] reported an overall MRSA contamination rate of 3.72% in meat and meat products, with higher prevalence in raw poultry (4.46%) and mixed livestock meats (3.86%). Alarmingly, regional estimates were even higher in parts of Asia and the Eastern Mediterranean, where pooled MRSA prevalence in meat exceeded 8–9%. In contrast, North American products demonstrated relatively lower contamination levels (1.89%)—a disparity largely attributed to stronger regulatory oversight and surveillance systems. Retail surveillance studies further confirm this trend. In China, several studies have documented MRSA presence in retail raw chicken, with prevalence estimates around [149]. Similarly, recent surveillance in pork demonstrated that fresh pork retailed in veterinary settings carried MRSA strains [150]. In southern Italy, Basanisi et al. [151] found an MRSA prevalence of 2.4% across 500 retail meat samples—including pork, poultry, and beef—indicating a lower but still significant regional burden compared to global estimates. These findings underscore the urgent need for improved hazard analysis and critical control point (HACCP) strategies during slaughter, processing, and packaging.

Critical intervention points include the prevention of direct contact between contaminated carcasses and clean equipment, routine screening of food handlers, and the implementation of effective sanitation protocols. Additionally, targeted molecular surveillance of MRSA clonal complexes circulating in abattoirs and retail markets can aid in identifying high-risk transmission routes, facilitating timely and evidence-based mitigation measures. Ultimately, minimizing post-harvest contamination is essential to safeguarding consumers and interrupting the zoonotic transmission of AMR staphylococci through the food chain.

### 4.3. Retail and Consumer Exposure: Raw Products, Undercooked Meats, and Poor Hygiene

At the final stage of the farm-to-fork continuum, consumers become vulnerable to AMR *Staphylococcus* species through various exposure routes—particularly via handling or consuming contaminated animal-derived foods. Raw or undercooked products such as unpasteurized milk, artisanal cheeses, and inadequately cooked meats serve as reservoirs for MRSA and other multidrug-resistant staphylococci. Inadequate food handling practices—including poor hand hygiene, the use of contaminated utensils without proper cleaning, and cross-contact between raw and ready-to-eat foods—significantly heighten household transmission risks [152].

Recent global meta-analyses have estimated that the prevalence of MRSA in retail meat ranges from 2% to over 8%, depending on the geographical region, animal species, and hygiene standards in meat processing and packaging facilities [148]. In Saudi Arabia, MRSA has been detected in approximately 20% of retail camel meat samples—as well as lower rates in other meats—where isolates carried the mecA gene, prompting concern over consumer exposure to clinically significant AMR determinants [153]. Similar contamination levels have been reported in other regions, including Europe and Asia, suggesting widespread dissemination across the global food supply chain.

Contamination is not limited to raw products. RTE foods have also emerged as a significant vehicle for enterotoxigenic and resistant *S. aureus*. In Algeria, Fanelli et al. [154] detected *S. aureus* in 23.2% of RTE samples, including sandwiches, cooked meats, and pastries. Molecular screening revealed the presence of classic enterotoxin genes (*sea*, *seb*, *see*) as well as multidrug resistance profiles, highlighting the risk of toxin-mediated foodborne illness in addition to AMR. Beyond food products, household environments serve as secondary reservoirs that facilitate persistent contamination. Cutting boards, kitchen sinks, refrigerators, and storage containers have all been identified as hotspots for resistant bacteria. Studies have shown that *S. aureus* can persist on plastic and wooden cutting boards even after cleaning, particularly in the presence of knife grooves and porous surfaces that retain moisture and organic material [155].

Intra-household transmission through contaminated surfaces poses a particular threat in settings with immunocompromised individuals or young children, where infections may be more severe. These findings underscore the need for enhanced consumer education regarding safe food handling, thorough cooking of animal products, and regular disinfection of food-contact surfaces. Integrating AMR risks into public food safety campaigns and surveillance of RTE and retail foods will be critical to reduce household exposure and break the cycle of zoonotic transmission.

### 4.4. One Health Implications and Risk Mitigation Strategies

The persistence and dissemination of AMR *Staphylococcus* spp. across the livestock production chain exemplify the core principles of the One Health paradigm, where microbial threats transcend species boundaries and environmental compartments. Addressing this challenge requires holistic, multisectoral interventions that span the entire farm-to-fork continuum. As livestock serve as both reservoirs and amplifiers of resistant bacteria, integrated risk mitigation must begin at the primary production level and extend through food processing, distribution, and consumer engagement. At the farm level, the implementation of strict biosecurity protocols—including control of animal movement, housing sanitation, and limitation of external vectors—has been consistently associated with reduced AMU and lower AMR prevalence. Enhanced mastitis control programs, incorporating regular somatic cell count monitoring, targeted antimicrobial therapy, and the use of effective vaccines against *Staphylococcus aureus*, have demonstrated success in reducing intramammary infections and the need for routine antibiotic administration [156]. Moreover, AMS initiatives that restrict the use of critically important antibiotics and promote veterinary oversight have proven effective in curbing inappropriate usage patterns, particularly in regions where non-therapeutic use remains prevalent [157].

In food processing environments, cross-contamination during slaughter, carcass dressing, and packaging presents a major conduit for AMR transmission. The adoption of good manufacturing practices, routine environmental microbiological monitoring, and worker hygiene training are critical to minimizing microbial loads on food-contact surfaces and in final meat products. Structural interventions—such as the segregation of clean and dirty zones, use of dedicated equipment for raw and RTE products, and rapid chilling technologies—can further interrupt transmission pathways. Studies have shown that HACCP-based protocols significantly reduce AMR bacterial presence in meat products when rigorously implemented [158]. At the retail and consumer level, public awareness and education are fundamental. The risks associated with consuming raw animal products—including unpasteurized milk, artisanal cheeses, and undercooked meat—must be clearly communicated to consumers through labeling, outreach, and dietary guidelines. Proper food handling practices in domestic settings—including handwashing, separation of raw and cooked foods, and adequate cleaning of utensils and kitchen surfaces—are essential to preventing household transmission of MRSA and other resistant staphylococci. Long-standing findings indicate that *S. aureus* and MRSA can persist on cutting boards and sinks, facilitating intra-household spread when hygiene practices are poor [155].

From a policy and surveillance standpoint, integrated One Health systems that unify veterinary, food safety, environmental, and public health data are essential for detecting and mitigating emerging AMR threats. Cross-sectoral monitoring platforms help trace resistance gene flow and inform targeted interventions. Policies limiting antimicrobial and co-selective agent use, alongside investments in biosecurity, can significantly reduce AMR prevalence. Alternatives such as probiotics, bacteriophages, and precision livestock tools also support productivity while minimizing resistance pressures [56]. Ultimately, a coordinated One Health approach—spanning farm practices, processing hygiene, consumer behavior, and regulation—is vital to curb AMR *Staphylococcus* spp. and safeguard antimicrobial efficacy in both human and animal health.

**Figure 3:**
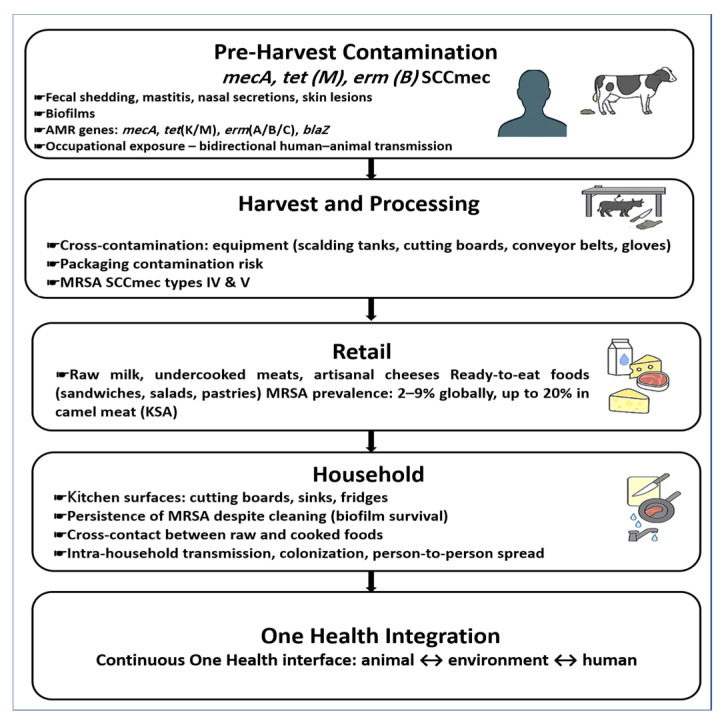
Farm-to-fork transmission pathways of AMR *Staphylococcus* spp. Antimicrobial-resistant *Staphylococcus* species spread along the farm-to-fork continuum, with risk factors at each stage including pre-harvest reservoirs (fecal shedding, mastitis, biofilms); slaughter and processing contamination (equipment, surfaces, packaging); retail products (raw milk, undercooked meats, ready-to-eat foods); and household exposure (improper handling, surface persistence, cross-contact).

As illustrated in Figure 3, the dissemination of antimicrobial-resistant *Staphylococcus* spp. follows a farm-to-fork continuum that begins with pre-harvest reservoirs such as fecal shedding, mastitis, and biofilm formation. During slaughter and processing, resistant strains are amplified through contaminated equipment, surfaces, and packaging, while retail foods—including raw milk, undercooked meats, and ready-to-eat products—serve as important transmission vehicles. At the household level, improper food handling, inadequate cooking, persistent contamination of kitchen surfaces, and intra-household spread further heighten the risk of exposure. These pathways demonstrate the interconnected One Health nature of AMR transmission across animal, environmental, and human domains.

## 5. Detection and Surveillance Tools for AMR Pathogens

Effective surveillance of AMR in livestock production requires a multi-tiered approach that integrates conventional microbiological assays, molecular diagnostics, and advanced genomic technologies. These tools serve as critical components in monitoring resistance patterns, identifying high-risk clones, and informing mitigation strategies across the farm-to-fork continuum. However, disparities in technological access, diagnostic capacity, and data integration hinder global harmonization and timely response.

### 5.1. Phenotypic Detection Methods

Phenotypic antimicrobial susceptibility testing (AST) remains the cornerstone of AMR surveillance due to its standardization, accessibility, and global regulatory acceptance. The disk diffusion method (Kirby–Bauer) and broth microdilution assays, as endorsed by the Clinical and Laboratory Standards Institute (CLSI) and the European Committee on Antimicrobial Susceptibility Testing (EUCAST), are the most widely used methodologies for determining minimum inhibitory concentrations (MICs) of bacterial isolates from livestock, food, and environmental samples [8]. These tests enable classification of pathogens as susceptible, intermediate, or resistant, serving as essential diagnostic tools in both veterinary and public health laboratories. However, traditional phenotypic methods are inherently limited by their reliance on viable, culturable bacteria and the need for incubation periods of 18–24 h. These constraints hinder their effectiveness in polymicrobial matrices, slow diagnostic workflows, and preclude real-time treatment guidance, especially in acute veterinary care and farm outbreak scenarios [2,159]. In response, several rapid phenotypic AST (rAST) technologies have emerged with the goal of reducing diagnostic turnaround without compromising accuracy. One such innovation is optical AST using deep-learning-enhanced imaging systems. Brown et al. [160] reported a novel microscopy-based AST platform that analyzes bacterial growth dynamics within 6–7 h, achieving over 95% categorical agreement with conventional broth microdilution. Similarly, single-cell Raman spectroscopy has enabled metabolic profiling of individual bacterial cells using deuterium-labeled water (D_2_O), providing MIC results within 2–3 h and accurately identifying resistance phenotypes [161].

Further advancements include surface-enhanced Raman scattering (SERS), which detects purine metabolite signatures as biomarkers of bacterial activity and drug susceptibility. Li et al. [162] demonstrated that SERS-based assays could classify resistant and susceptible phenotypes across Gram-positive and Gram-negative species in approximately one hour. Additionally, machine learning-assisted phenotyping systems now leverage time-lapse imaging and morphometric analysis to distinguish susceptible from resistant strains in under 30 min [163]. Despite their promise, these cutting-edge methods are still largely confined to research settings. Their deployment in field-based veterinary contexts is constrained by high costs, infrastructure needs, and limited regulatory validation. Moreover, standardization across bacterial species, antimicrobial panels, and livestock matrices remains under development. Until such tools are fully validated and integrated into routine diagnostic pipelines, traditional phenotypic AST will continue to serve as the benchmark for AMR surveillance in primary animal production.

### 5.2. Molecular Diagnostics

Molecular diagnostic techniques have revolutionized AMR surveillance by offering rapid, sensitive, and specific detection of resistance genes in both culturable and non-culturable organisms. These methods are now widely used across veterinary, environmental, and food production sectors to complement or replace conventional phenotypic assays, particularly when rapid decision-making is essential. PCR and quantitative PCR (qPCR) remain the most commonly applied molecular tools for AMR detection due to their robustness, scalability, and ability to target specific genetic markers. These assays enable the identification of a wide range of resistance determinants, including *mecA*, *bla_CTX-M*, *ermB*, and *tetM*, in DNA extracted from pure isolates, feces, carcasses, milk, and environmental matrices. For example, Roschanski et al. [164] developed a multiplex real-time PCR assay that concurrently detects the predominant β-lactamase genes bla_CTX-M, bla_TEM, and bla_SHV, achieving limits of detection around 10–20 gene copies/µL and enabling results in under 3 h. Additionally, Velasco et al. [165] validated a multiplex qPCR for simultaneous detection of *nuc* and mecA genes in *S. aureus* from animal and meat samples, demonstrating robust detection after enrichment and providing results within ~6–8 h, significantly reducing diagnostic time. However, molecular diagnostics also face important limitations, including high costs, the need for skilled personnel, challenges in field applicability for low-resource settings, and risks of false negatives due to PCR inhibitors or low DNA quality [166,167].

LAMP has emerged as a powerful alternative for AMR gene detection in low-resource and field-based settings. Unlike PCR, LAMP operates at a single temperature and does not require a thermocycler, making it particularly suitable for on-farm use and rapid screening. Cui et al. [168] developed a colorimetric LAMP assay for *E. coli* O157:H7 detection in milk, achieving detection within 45 min with a visual color change readout. Similarly, Long et al. [169] validated LAMP for the detection of *S. aureus* in veterinary samples, reporting pooled sensitivity and specificity above 98%. The integration of LAMP with immunocapture and magnetic bead separation has further expanded its applicability for multiplex detection of major zoonotic bacteria such as *S. enterica*, *S. aureus*, and *E. coli*, with a total assay time of under 90 min.

CRISPR-based diagnostics represent a newer frontier in molecular AMR surveillance. These systems utilize CRISPR-associated (Cas) enzymes programmed with guide RNAs to recognize and cleave target DNA sequences with high specificity. Diagnostic platforms such as SHERLOCK (Specific High Sensitivity Enzymatic Reporter UnLOCKing) and DETECTR (DNA Endonuclease Targeted CRISPR Trans Reporter) have shown the potential to detect AMR genes such as *mcr-1*, *bla_NDM*, and *tetM* in clinical and foodborne bacteria. Chertow [170] reported the development of lateral-flow and fluorescence-based CRISPR assays capable of detecting resistance genes at attomolar concentrations. Although CRISPR diagnostics are not yet widely deployed in veterinary practice, ongoing advancements in point-of-care device miniaturization and sample preparation workflows are likely to accelerate their field applicability in the coming years. Collectively, molecular diagnostics enable faster, more accurate AMR surveillance across the livestock production continuum, supporting early outbreak detection, targeted antimicrobial use, and improved biosecurity strategies.

### 5.3. Advanced Genomic and Proteomic Technologies

The integration of advanced genomic and proteomic tools into AMR surveillance has markedly expanded the scope, resolution, and accuracy of resistance detection across livestock, food, and environmental settings. These technologies not only facilitate comprehensive profiling of resistance determinants but also enable source tracking and real-time epidemiological monitoring, particularly within One Health frameworks. WGS has emerged as a transformative tool for AMR surveillance by enabling the precise identification of resistance genes, MGEs, and clonal relationships among bacterial populations. WGS offers unparalleled resolution in detecting chromosomal mutations, plasmid-borne resistance determinants, and horizontal gene transfer events, which are crucial for understanding the evolution and dissemination of MDR pathogens. National and international surveillance initiatives—including those led by the European Union Reference Laboratory for AMR—have begun integrating WGS into routine workflows to support outbreak investigations, source attribution, and risk assessments [171,172]. Despite these advantages, the broad application of WGS in LMICs is constrained by high costs, limited sequencing infrastructure, and the need for advanced bioinformatics expertise [38].

Metagenomic sequencing, particularly shotgun metagenomics, provides a culture-independent approach to AMR detection by capturing the total resistome within complex matrices such as feces, soil, wastewater, and food products. This technique enables untargeted profiling of both known and novel resistance genes, including those harbored by uncultivable or rare bacterial taxa. Recent studies have employed metagenomics to detect ARGs conferring resistance to tetracyclines, sulfonamides, and β-lactams in livestock environments, highlighting the role of MGEs—such as integrons, transposons, and plasmids—in resistance dissemination [14,173]. However, implementation challenges persist due to high sequencing depth requirements, data complexity, and the need for standardized analytical pipelines across laboratories. MALDI-TOF MS is now routinely used in veterinary microbiology laboratories for rapid and cost-effective bacterial identification at the genus and species levels [174].

While traditionally limited to taxonomic classification, MALDI-TOF MS is increasingly being adapted for resistance detection when coupled with functional assays, such as β-lactamase hydrolysis tests and PCR-based resistance gene identification. Recent innovations have demonstrated the feasibility of integrating MALDI-TOF MS with resistance phenotype prediction algorithms and ESBL detection protocols in *S. aureus*, *E. coli*, and other livestock-associated pathogens [175]. These advancements enhance diagnostic turnaround and complement molecular and culture-based approaches in AMR monitoring systems. In summary, WGS, metagenomics, and MALDI-TOF MS offer complementary capabilities that are reshaping AMR detection in veterinary and food microbiology. Their continued integration into surveillance programs requires strategic investment in infrastructure, training, and international data harmonization to ensure equitable access and effective implementation across regions.

**Figure 4:**
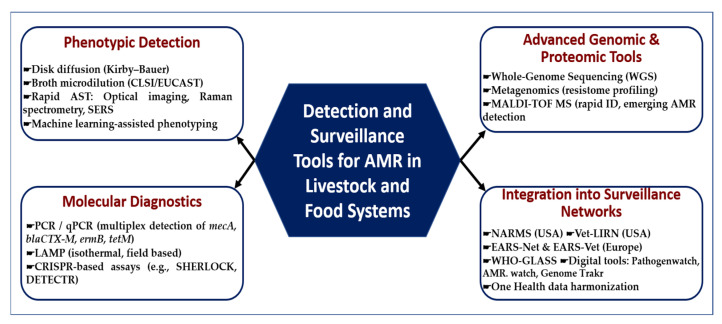
Detection and surveillance tools for AMR in livestock and food systems. This framework summarizes the multi-tiered tools used to detect, characterize, and monitor AMR across livestock and food chains. Approaches include phenotypic detection (disk diffusion, broth microdilution, rapid AST, machine learning-assisted phenotyping); molecular diagnostics (PCR/qPCR, LAMP, CRISPR-based assays); advanced genomic and proteomic technologies (WGS, metagenomics, MALDI-TOF MS); and integration into surveillance networks (NARMS, Vet-LIRN, EARS-Net, WHO-GLASS, and digital platforms such as Pathogenwatch and GenomeTrakr).

As shown in Figure 4, the detection and surveillance of AMR in livestock and food systems relies on a complementary toolbox that spans from classical to cutting-edge technologies. Phenotypic assays such as disk diffusion and broth microdilution remain the global reference standards, while rapid optical and machine learning-based methods are emerging to accelerate turnaround times. Molecular diagnostics—including multiplex PCR/qPCR, isothermal LAMP assays, and CRISPR-based platforms—offer high sensitivity for gene-level detection. At a higher resolution, advanced genomic and proteomic tools such as whole-genome sequencing, metagenomics, and MALDI-TOF MS enable comprehensive profiling of resistance determinants and bacterial lineages. Critically, these methods are being embedded into global surveillance networks (NARMS, Vet-LIRN, EARS-Net, WHO-GLASS) and digital platforms that facilitate One Health integration and cross-sectoral data harmonization.

### 5.4. Integration into Surveillance Networks

The global response to AMR has increasingly emphasized the importance of integrated, multisectoral surveillance systems grounded in the One Health paradigm. These systems aim to consolidate AMR data across human, veterinary, food, and environmental domains to enable coordinated interventions and informed policymaking. One of the most well-established surveillance frameworks is the National AMR Monitoring System (NARMS) in the United States. Founded in 1996 through a collaboration among the Centre for Disease Prevention and Control (CDC), Food and Drug Administration (FDA), and U.S. Department of Agriculture (USDA), NARMS monitors AMR trends in enteric bacteria from humans, retail meats, and food animals. The program integrates phenotypic and genotypic methods, including WGS, and supports the early detection of emerging resistance threats across the food chain [176,177]. Complementing NARMS, the Veterinary Laboratory Investigation and Response Network (Vet-LIRN) coordinates with veterinary diagnostic laboratories across the United States to standardize AST and expand WGS-based surveillance of key veterinary pathogens, such as *E. coli*, *S. enterica*, and *S. pseudintermedius* [178].

In Europe, the European AMR Surveillance Network (EARS-Net) monitors resistance in human clinical isolates, while the European AMR Surveillance Network in Veterinary Medicine (EARS-Vet) extends this scope to veterinary pathogens and zoonotic bacteria. Both networks aim to harmonize AST methodologies and enable coordinated risk assessments across member states [179]. Developed under the EU-JAMRAI initiative, EARS-Vet supports the collection of resistance data from major veterinary pathogens and aligns with EARS-Net to facilitate One Health integration and evidence-based policy on AMU in animals [180].

Standardizing data integration between human and veterinary AMR surveillance requires harmonized AST protocols. Aligning CLSI and EUCAST breakpoints, adopting ISO-based laboratory standards, and implementing shared quality-control schemes are critical to ensure comparability of results across sectors. Equally important are interoperable databases that link veterinary and human AMR networks, such as WHO-GLASS, EARS-Net, NARMS, and the WOAH/FAO/WHO Tricycle project, which demonstrate how methodological differences can be bridged under a One Health framework [17,181].

Despite these advances, major implementation gaps remain, particularly in LMICs. For example, a pan-African assessment revealed that among more than 53,000 laboratories across 14 sub-Saharan African countries, only about 1% were equipped to perform bacteriology and AST, limiting AMR detection capacity [182]. In Vietnam, surveillance of poultry and swine reported over 90% of *E. coli* and non-typhoidal *Salmonella* isolates as multidrug-resistant, highlighting severe monitoring limitations [183]. Challenges such as inadequate laboratory infrastructure, shortage of trained personnel, and limited access to molecular diagnostics further hinder comprehensive AMR surveillance. To address these disparities, the One Health Joint Plan of Action (2022–2026), developed collaboratively by the WHO, FAO, WOAH, and UNEP, outlines strategic objectives for capacity building, data harmonization, and multisectoral collaboration [19]. The plan emphasizes the integration of AMR data from human, animal, and environmental sectors to improve situational awareness and global response strategies.

Emerging digital platforms are playing a transformative role in global AMR surveillance by enabling genome-based analyses for real-time insights. Pathogenwatch and AMR.watch offer user-friendly web interfaces for WGS data upload, resistance gene prediction, and phylogenetic placement of bacterial genomes—facilitating applications in both public health and veterinary microbiology [184,185]. Similarly, the GenomeTrakr network, maintained by the U.S. FDA, aggregates WGS data for foodborne pathogens from global sources, while the National Database of Antibiotic Resistant Organisms hosted by NCBI serves as a comprehensive repository of resistance genotypes and metadata [177]. Other resources such as AMRmap and Resistome Tracker offer visualization of resistance gene distributions by geography, microbial taxonomy, and sample type, enhancing surveillance transparency and facilitating international data sharing. Moving forward, equitable AMR surveillance will depend on the implementation of tiered diagnostic frameworks that combine portable field-based tools—such as LAMP and qPCR—with centralized genomic and proteomic technologies. International investment in training, infrastructure, and governance frameworks will be essential to ensure inclusive participation and sustainable surveillance coverage across all regions.

### 5.5. Comparative Utility of AMR Detection Tools

A comparative overview of AMR detection methods used across the livestock food production continuum is presented in Table 3. Each method is evaluated based on key performance characteristics, including detection speed, sensitivity, scalability, and field applicability, to assess its suitability in farm, processing, and clinical settings. The table also highlights method-specific limitations and provides representative references supporting their use in One Health surveillance frameworks. This comparative approach is essential for guiding stakeholders in selecting context-appropriate diagnostic tools to enhance AMR monitoring and mitigation strategies.

## 6. Regional and Global Epidemiological Trends

AMR in livestock production exhibits pronounced geographic heterogeneity, shaped by differences in farming intensity, AMU practices, and national surveillance capacity. Regional disparities in monitoring and reporting have led to uneven global understanding, with several high-burden areas remaining underrepresented. This section outlines key AMR patterns in livestock across major world regions and highlights the global dissemination of priority resistance genes, including *mcr*, *blaNDM*, and *tetX*, while also identifying persistent data gaps that hinder One Health-oriented containment strategies.

Europe, particularly the EU, benefits from robust, harmonized AMR surveillance systems coordinated by the European Food Safety Authority (EFSA) and the European Centre for Disease Prevention and Control (ECDC). The latest EFSA/ECDC joint report [188] confirms high levels of resistance in Campylobacter from broilers and the widespread presence of ESBL/AmpC-producing *E. coli* in poultry and pigs. Notably, livestock-associated *S. aureus* ST398 continues to dominate pig farms across several member states. In Denmark, for example, ST398 accounted for 23% of all new MRSA infections in 2023 [189], demonstrating its public health significance. Pan-European data further indicate MRSA colonization in over 75% of pig farms [190], emphasizing the importance of occupational exposure and environmental persistence in transmission dynamics.

In the Middle East and North Africa regions, systematic AMR surveillance in livestock remains limited. Nonetheless, targeted studies in countries such as Saudi Arabia and Egypt report concerning levels of ESBL-producing *E. coli*, multidrug-resistant *Salmonella*, and MRSA (including ST398) in poultry and dairy sectors [9,191]. While these findings underscore the zoonotic potential of resistant pathogens, the absence of coordinated One Health frameworks impedes accurate burden estimation and timely policy response. Most nations in this region lack integrated data platforms capable of tracking AMR trends across animal, food, and human health sectors [20].

Asia, particularly China, India, and Southeast Asia, represents a global hotspot for AMR gene emergence and dissemination. Intensive livestock production systems with high antimicrobial usage have fostered the expansion of plasmid-mediated resistance determinants. In China, for instance, the *mcr-1* gene—conferring colistin resistance—has become entrenched in *E. coli* from swine and poultry [40,41]. Concurrently, the *blaNDM* gene has rapidly expanded through the food chain—from animal farms to retail meat—driven in China by the dissemination of *blaNDM-5* carrying IncX3 plasmids in carbapenemase-producing *E. coli* isolated from retail meat products, with prevalence rising from ~9% in 2016 to >22% by 2018 [192]. Of particular concern is the emergence of the *tet(X4)* gene—conferring tigecycline resistance—which has been detected in *E. coli* from pig farms, occasionally co-occurring with *mcr-1* on mobile plasmids [193]. Recent surveys in Hong Kong and Southeast Asia further report high levels of ESBL- and *mcr*-positive *E. coli* in retail meat, indicating significant consumer exposure risk [194]. These trends highlight the urgent need for integrated surveillance and AMS in the region.

Sub-Saharan Africa remains one of the most under-documented regions regarding AMR in food-producing animals, despite accumulating evidence of significant risk. Although the prevalence of MRSA in livestock is generally low—typically below 3%—sporadic reports from Senegal, Nigeria, and South Africa confirm its presence in pigs and cattle [195]. In contrast, high rates of ESBL-producing *E. coli* and *K. pneumoniae* have been reported in poultry, dairy, and aquaculture systems across West, East, and North Africa. Studies from Ghana, Nigeria, and Tunisia consistently identify *blaCTX-M-15*, *sul1*, and *tetA* as the most frequently detected resistance genes, often located on MGEs with zoonotic potential [66,67]. Furthermore, sporadic detection of carbapenemase genes such as *blaOXA-48*, *NDM*, and *VIM* in animal and environmental samples underscores the risk of further resistance escalation [68]. However, persistent limitations in diagnostic infrastructure, genomic surveillance capacity, and intersectoral data integration continue to hinder comprehensive AMR monitoring and One Health-based policy responses across the region.

MGEs, including plasmids, integrons, and transposons, have played a pivotal role in the global dissemination of critical AMR genes across livestock-associated bacterial populations. The *mcr* gene family, first identified in *E. coli* from pigs in China in 2015, has since been detected in food animal isolates on every continent and in multiple genera, including *Salmonella*, *Klebsiella*, and *Enterobacter* [40,196], has now been detected in food animal isolates on every continent. Similarly, the *blaNDM* gene, encoding New Delhi metallo-β-lactamase, has now been reported in over 60 bacterial species worldwide, including zoonotic and foodborne pathogens, despite the absence of approved carbapenem use in food-producing animals [197]. Of growing concern is the *tetX* gene family, particularly *tetX4*, which confers resistance to tigecycline—a last-resort antibiotic—and has rapidly spread across Asia, primarily in *E. coli* from swine and poultry [198,199]. These resistance genes are frequently embedded in conjugative plasmids co-harboring multiple determinants, facilitating horizontal transfer between species and accelerating the evolution of multidrug-resistant pathogens in animal production systems [35,200].

Despite notable progress in regions such as the European Union, North America, and parts of East Asia, global AMR surveillance in livestock remains fragmented and uneven. Comprehensive, real-time genomic monitoring is largely confined to high-income countries, while many low- and middle-income regions—including large parts of Africa, the Middle East, and South Asia—lack sustained infrastructure for AMR detection, reporting, and data integration. Recognizing these disparities, the Quadripartite One Health Joint Plan of Action (2022–2026), developed collaboratively by the WHO, the FAO, the WOAH, and the UNEP, outlines strategic priorities to enhance AMR surveillance through tiered diagnostic frameworks, centralized data platforms, and cross-sectoral coordination [19]. The WOAH’s 2024 global progress report further underscores persistent gaps in veterinary laboratory capacity, weak linkages between animal and human AMR data systems, and underinvestment in environmental monitoring [201]. Without targeted investments in diagnostic equity and integrated surveillance infrastructure, the global community risks overlooking early signals of emerging resistance, thereby undermining timely public health responses and containment strategies.

## 7. Public Health Implications and One Health Integration

### 7.1. Foodborne Illness Burden from AMR Pathogens

Food-producing animals are an important source of AMR bacteria that reach humans through contaminated meat, dairy, eggs, and cross-contaminated kitchen environments. Yet, despite clear mechanistic links, the quantitative contribution of food to the overall human AMR burden remains poorly resolved. A 2010–2023 systematic review concluded that the human health burden attributable specifically to foodborne AMR cannot presently be robustly estimated because of heterogeneous study designs, limited integration of genomic data, and sparse exposure assessment. Meanwhile, global all-cause AMR mortality estimates (1.27 million deaths attributable; 4.95 million associated in 2019) highlight the scale of the problem but do not partition the foodborne component. EFSA/ECDC and recent syntheses nevertheless indicate substantial resistance in *Salmonella*, *Campylobacter*, and *E. coli* from the food chain, underscoring the plausibility of a meaningful foodborne contribution [191,202].

### 7.2. Human Colonization with Livestock-Associated Strains

Humans frequently acquire resistant livestock-associated strains through direct or indirect animal contact. Livestock-associated MRSA (LA-MRSA) ST398 exemplifies this dynamic; meta-analyses show markedly elevated colonization in livestock workers compared with the general population (≈14% vs. 0.8–1.3%), and farm-based studies report colonization in more than half of pig workers, with virtually all isolates belonging to ST398. Persistent environmental reservoirs (e.g., barn dust) further sustain transmission pressure. Such colonization can act as a silent reservoir that seeds subsequent infections or facilitates onward transmission into the community [203,204].

### 7.3. Occupational Risks (Farmers, Veterinarians, Slaughterhouse Workers)

Occupationally exposed groups—farmers, veterinarians, animal transporters, and slaughterhouse workers—consistently demonstrate higher carriage and occasionally infection rates with LA-MRSA, ESBL-producing Enterobacterales, and fluoroquinolone-resistant *Campylobacter*. These workers can function as bridging hosts, introducing resistant strains into households, healthcare facilities, and the broader community, highlighting the need for tailored preventive measures (e.g., targeted screening, hygiene protocols, and decoupling high-intensity animal contact from hospital work when feasible) [203].

### 7.4. Resistance Spillover into Human Hospitals (e.g., ST398 MRSA)

Spillover from livestock into hospitals is now well documented. In Denmark and other European settings, livestock-associated MRSA *ST398* infections (including bloodstream infections) rose in parallel with the expansion of pig production, and genome-based studies have demonstrated close relatedness between human clinical and porcine isolates. These data confirm that livestock-origin strains such as *S. aureus ST398* can adapt, persist, and cause serious disease in humans, challenging the historical notion that LA-MRSA is primarily a colonizer of limited clinical relevance [205,206].

### 7.5. One Health Barriers in LMICs and the Need for Integrated Antimicrobial Stewardship

Despite strong policy rhetoric, operational One Health integration remains uneven, particularly in LMICs. Key barriers include (i) fragmented governance and siloed budgets across health, agriculture, and environment; (ii) limited laboratory and genomic capacity to detect and track AMR in animal and environmental sectors; (iii) poor interoperability of data systems; and (iv) underregulated antimicrobial access and use in livestock. The Quadripartite One Health Joint Plan of Action (2022–2026) calls for tiered diagnostic frameworks, centralized and interoperable data platforms, and routine integration of animal and environmental AMR indicators into national action plans—priorities echoed and operationalized in WOAH’s 2024 AMR progress report, which documents persistent capacity gaps and variable engagement with global platforms (e.g., ANIMUSE). A recent scoping review of One Health implementation similarly highlights governance, financing, and data-sharing deficits as recurrent obstacles [19,207].

To translate One Health principles into effective action in LMICs, a phased and pragmatic approach to AMS is essential. Building tiered diagnostic systems should begin with the deployment of affordable, field-ready tools such as LAMP and qPCR at peripheral levels, linked to centralized reference laboratories capable of WGS and metagenomics for outbreak investigation and resistance tracking. Simultaneously, it is crucial to establish interoperable data systems that integrate AMR and AMU data from the animal and environmental sectors into national surveillance dashboards aligned with human health systems. Cross-sectoral AMS efforts must include the harmonization of CIA restriction lists, enforce veterinary prescription auditing and dispensing regulations, and incentivize sustainable alternatives at the farm level, such as vaccines, probiotics, bacteriophage therapy, and precision husbandry practices. Occupational health protections for high-risk groups—such as farmers, veterinarians, and slaughterhouse workers—should be strengthened through targeted screening, decolonization protocols, and improved on-farm biosecurity and ventilation to reduce environmental reservoirs like dust-borne livestock-associated MRSA. Lastly, sustainable implementation demands that One Health AMR initiatives be integrated into national budgets and governance structures, moving beyond donor-dependent pilot projects to establish shared accountability indicators across health, agriculture, and environmental ministries. Without such coordinated investments and institutional commitments, LMICs remain vulnerable to undetected emergence and spread of resistance—particularly plasmid-mediated genes in livestock—undermining timely public health responses and exacerbating global AMR burdens [19,207].

**Figure 5:**
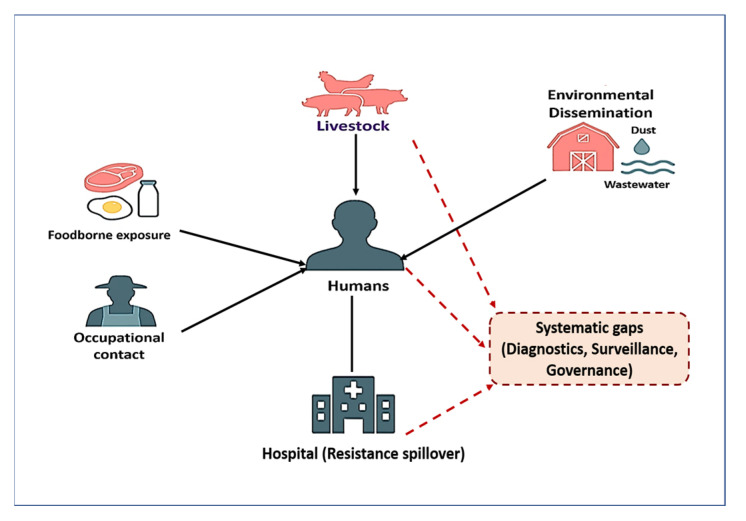
One Health transmission pathways of AMR bacteria originating from livestock. Solid black arrows indicate established pathways: foodborne exposure, occupational contact, environmental dissemination (manure, wastewater, dust), and resistance spillover into hospitals. Dashed red arrows and the shaded red box denote systemic gaps in surveillance, diagnostics, and governance frameworks—particularly in low- and middle-income countries—that hinder timely AMR containment.

Figure 5 illustrates the major zoonotic, foodborne, and environmental transmission pathways of AMR, while highlighting gaps in surveillance, diagnostics, and governance frameworks. Dashed red arrows and the shaded red box represent these deficiencies, particularly in LMICs, ensuring that the schematic conveys both transmission pathways and systemic gaps. Table 4 summarizes key public health risks associated with AMR in livestock production systems and outlines systemic One Health barriers, particularly in LMICs. It highlights foodborne transmission, occupational exposure, human colonization, and spillover into healthcare settings, alongside critical gaps in governance, diagnostics, and stewardship infrastructure that hinder AMR containment across the animal–human–environment interface.

## 8. Mitigation Strategies and Innovation

Mitigating AMR in livestock requires an integrated and multifaceted strategy encompassing biological alternatives, farm-level interventions, digital innovations, and effective regulatory frameworks. Among biological alternatives, vaccines, bacteriophages, probiotics, prebiotics, synbiotics, and organic acids are increasingly employed to reduce pathogen load and AMU. Targeted vaccination against pathogens such as *Salmonella* and *E. coli* has demonstrated efficacy in lowering disease incidence and consequently reducing AMU. Bacteriophage therapy—routinely used in countries like Georgia and parts of Eastern Europe—offers highly specific lytic activity against bacterial pathogens without inducing off-target resistance. Likewise, probiotics and prebiotics enhance gastrointestinal health and modulate immune function, thereby reducing infection pressure and offering a sustainable alternative to antibiotic growth promoters in animal husbandry systems [213].

At the farm level, critical interventions include strengthened biosecurity measures, improved hygiene practices, the use of rapid diagnostics to guide targeted antimicrobial therapy, strict adherence to drug withdrawal periods, and continuous veterinary training. A notable example is Denmark’s Yellow Card initiative, which, through benchmarking and monitoring of farm-level AMU, achieved a sustained 25–28% reduction in antimicrobial use per pig without compromising herd health or productivity [214]. Emerging precision agriculture and artificial intelligence (AI) technologies now enable real-time AMR surveillance and risk forecasting. Machine learning (ML) algorithms trained on metagenomic data and environmental sensor inputs have been successfully applied to poultry systems, allowing early prediction of *E. coli* resistance patterns and guiding timely, site-specific interventions. These tools represent a promising advancement for optimizing AMU and enhancing disease prevention strategies through predictive analytics [215].

On the regulatory and governance front, a range of international frameworks—including the Codex Alimentarius guidelines on AMR, the European Union’s ban on antibiotic growth promoters, and national surveillance systems like Denmark’s VetStat—have set important global precedents. Nonetheless, the implementation and enforcement of such policies remain inconsistent, particularly across LMICs. Challenges include limited institutional capacity, insufficient surveillance infrastructure, and fragmented policy alignment [214]. Denmark’s Yellow Card system, introduced in 2010, stands out as a scalable, evidence-based model; by setting AMU thresholds and mandating corrective action when exceeded, it has effectively reduced usage, particularly in high-consumption farms, and has positioned Denmark as a global exemplar in livestock AMR stewardship [216].

Table 5 provides a concise overview of evidence-based mitigation strategies to curb AMR in livestock production systems. It outlines core intervention domains—including biological alternatives, farm-level AMS, precision technologies, and regulatory frameworks—while highlighting exemplar practices, observed outcomes, and key implementation requirements. The table also features Denmark’s Yellow Card initiative as a case study for scalable stewardship, emphasizing its measurable impact on AMU reduction. This synthesis is particularly relevant for guiding practical, One Health-aligned AMR interventions in LMICs, where resource-sensitive approaches are critically needed.

## 9. Future Directions and Research Gaps

Despite the expanding array of mitigation strategies outlined in the previous section, several critical knowledge and implementation gaps continue to limit the effectiveness of AMR control in livestock systems. Addressing these gaps is essential to advance One Health-integrated responses, inform evidence-based policy, and future-proof surveillance and stewardship strategies across diverse production contexts. Combating AMR in livestock demands ongoing innovation, sustained investment, and cohesive cross-sectoral collaboration.

One of the most transformative tools for AMR monitoring is real-time genomic surveillance. Techniques such as WGS and metagenomics offer unprecedented resolution in tracing resistance evolution, pathogen transmission, and source attribution. However, the integration of these technologies into national AMR surveillance programs remains limited, particularly in LMICs, due to challenges related to cost, laboratory infrastructure, and data governance. Building regional sequencing hubs, enhancing bioinformatics capacity, and promoting the use of cloud-based platforms are crucial steps toward enabling real-time outbreak detection and resistance monitoring [220,221].

Artificial intelligence (AI) and machine learning (ML) are also emerging as powerful tools for predicting AMR patterns. Algorithms trained on WGS data, AMU trends, and environmental metadata have shown promise in forecasting resistance phenotypes. Tools such as DeepARG and PathoFact can identify both known and novel resistance genes, but their application in veterinary and livestock contexts remains very limited and requires rigorous validation across diverse ecological and geographic settings. This limitation highlights the urgent need for translational research to move AI/ML from proof-of-concept into practical One Health surveillance and decision-support platforms. Embedding AI-driven prediction models into digital farm management systems could further support proactive and precise antimicrobial stewardship in animal production [222].

A major obstacle to effective One Health AMR control is the lack of harmonized surveillance data systems. Information on AMR and AMU remains siloed across human, veterinary, and environmental sectors, precluding integrated analysis and rapid response. Initiatives such as the Quadripartite Integrated Surveillance System (QISS) and the WOAH’s ANIMUSE database aim to bridge these divides, yet global uptake remains slow, underscoring the urgent need for harmonization and integration of AMR data across sectors under the One Health framework. Greater international cooperation and standardized data-sharing protocols are critical to transforming these initiatives into globally functional surveillance networks.

Current research also tends to disproportionately target well-known pathogens such as *E. coli*, *Salmonella enterica*, and *S. aureus*, while neglected pathogens—including *Enterococcus* spp. and *Campylobacter coli*—receive limited attention. Moreover, under-researched production systems such as smallholder farms, pastoralist communities, and extensive grazing operations are rarely included in surveillance programs, particularly in LMICs. A broader focus is urgently needed to uncover hidden AMR reservoirs and better understand the socio-ecological determinants of resistance [212].

Finally, the emergence of novel protein production systems—including intensive aquaculture and insect farming—presents new challenges for AMR containment. Edible insect-rearing operations often involve high microbial densities and the use of feed additives, potentially fostering resistance gene amplification [223,224]. Likewise, aquaculture environments have been associated with rising AMR levels in pathogens such as *Aeromonas* spp., driven by routine antimicrobial use. These sectors remain poorly regulated and under-studied in the context of AMR, underscoring the need for targeted research and tailored stewardship frameworks [225].

**Figure 6:**
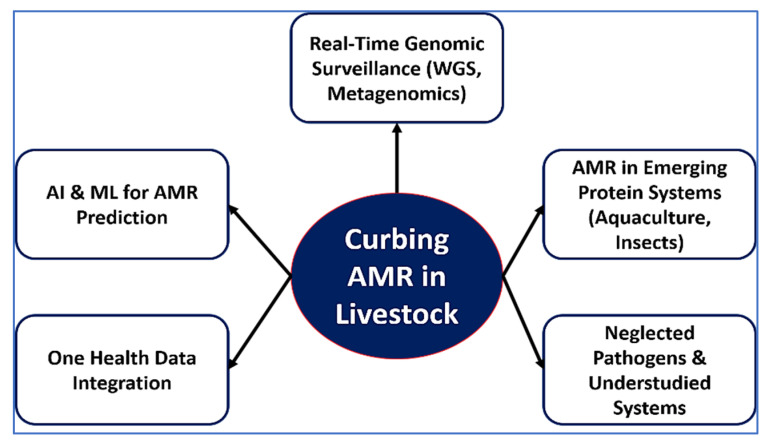
Emerging research priorities to curb AMR in livestock. The schematic highlights five critical frontiers for advancing AMR containment in livestock production systems: (1) real-time genomic surveillance using WGS and metagenomics, (2) AI and ML for AMR phenotype prediction and decision support, (3) harmonization of AMU/AMR data across One Health sectors, (4) targeted attention to neglected pathogens and under-researched production systems such as smallholder farms, and (5) emerging AMR risks associated with novel protein industries including aquaculture and insect farming.

Addressing the outlined research gaps—ranging from genomic surveillance and AI-based prediction to neglected pathogens, underrepresented production systems, and novel protein sources—will require coordinated global investment, policy innovation, and capacity building. These frontiers not only represent scientific priorities but also define the critical path toward safeguarding the efficacy of antimicrobials across the food chain. Without proactive efforts to close these knowledge gaps, the global community risks falling behind in the race against AMR. As summarized in Figure 6, the research and governance roadmap should prioritize five domains: the adoption of real-time genomic surveillance platforms, integration of AI/ML-based prediction tools into farm-level decision-making, harmonization of AMU/AMR data across One Health sectors, targeted surveillance of underrepresented pathogens and production systems, and assessment of AMR risks in emerging protein industries such as aquaculture and insect farming. Together, these interconnected priorities define the path forward to advance sustainable AMR containment under a One Health framework.

## 10. Conclusions

AMR in livestock is a multifaceted and escalating threat that bridges animal health, food safety, and global public health. This review has highlighted the complex interplay of factors driving AMR emergence in animal agriculture, including the widespread use of antimicrobials for growth promotion, therapeutic mismanagement, environmental contamination, and the unregulated exchange of mobile genetic elements. While intensive surveillance of major zoonotic pathogens such as *Salmonella*, *E. coli*, and *S. aureus* has yielded valuable insights, the persistence of resistance reservoirs in commensal flora, minor species, and under-monitored production systems remains a major blind spot in global AMR mitigation efforts. Innovative detection and monitoring strategies—including whole-genome sequencing, MALDI-TOF MS, and CRISPR-based diagnostics—offer transformative potential for real-time resistance tracking and targeted intervention. However, their implementation is still uneven, particularly in low- and middle-income countries, due to cost, infrastructure, and regulatory challenges. Similarly, antimicrobial stewardship remains fragmented, constrained by weak policy enforcement, limited veterinary oversight, and data silos across the One Health spectrum. To move from containment to control, future strategies must embrace cross-sectoral coordination, harmonized data systems, and scalable, context-sensitive stewardship programs. Prioritizing neglected pathogens, addressing AMR in emerging protein sectors (such as aquaculture and insect farming), and integrating artificial intelligence into predictive surveillance are no longer optional—they are imperatives for safeguarding both animal and human therapeutics. Ultimately, combating AMR in livestock demands not only scientific innovation but also political will, behavioral change, and sustained investment in global One Health infrastructure. The road ahead is complex, but failure to act decisively will compromise the effectiveness of modern medicine and the sustainability of food systems worldwide.

## Figures and Tables

**Table 1:** Global prevalence of selected ARGs in livestock-associated bacteria.

Resistance Gene	Major Hosts	Regions with High Prevalence	Key Notes	References
*blaCTX*-M	*E. coli* (poultry, cattle, swine)	Asia, Africa, Europe, Americas	Most widespread ESBL gene family; blaCTX-M-15 and blaCTX-M-14 dominate in livestock; disseminated via plasmids and integrons	[44,45]
*mcr-1*	*E. coli* (poultry, swine)	China, Southeast Asia, Latin America, Africa, Europe	First plasmid-mediated colistin resistance gene; pooled prevalence ~15–16% in chickens and pigs worldwide	[46,47]
*tetM*	Enterococcus, *S. aureus* (poultry, cattle)	Europe, North America, Africa	Most frequent transferable tetracycline resistance gene; associated with Tn916/Tn1545 transposons	[48,49]

**Table 2:** Overview of major AMR bacterial pathogens in livestock: resistance genes, host distribution, and zoonotic relevance.

Pathogen	Host Species	Key Resistance Genes	Clinical Relevance in Animals	Zoonotic Risk/Transmission Route	References
*E. coli* (STEC, ExPEC)	Poultry, Cattle, Swine	*bla_CTX-M*, *bla_SHV*, *mcr-1*	Enteritis, septicemia, urinary infections	Meat, feces, direct contact	[40,41]
*S. enterica*	Poultry, Swine	*bla_TEM*, *bla_CTX-M*, *qnrB*	Enteritis, systemic salmonellosis	Foodborne, fecal contamination	[43,83,84]
*C. jejuni*	Poultry	*gyrA*, *23S rRNA*, *tet(O)*	Asymptomatic in birds; diarrhea in animals	Undercooked meat, environment	[50,51]
*L. monocytogenes*	Dairy Cattle, Goats	*tetM*, *tetS*, *ermB*	Mastitis, encephalitis	Raw milk, cheese	[85,86]
*S. aureus* (LA-MRSA, ST398)	Pigs, Cattle, Poultry	*mecA*, *tetM*, *ermC*	Mastitis, wound infections	Occupational exposure, meat	[87,88,89]
*E. faecalis, E. faecium*	Swine, Poultry	*vanA*, *vanB*, *aac(6′)-Ie-aph(2″)-Ia*	Gut colonization, opportunistic infections	Manure, cross-species transmission	[66,90,91]
*K. pneumoniae*	Dairy Cattle, Poultry	*bla_KPC*, *bla_OXA-48*, *mcr-1*	Mastitis, respiratory infections	Contaminated milk, nosocomial potential	[92]
*S. pseudintermedius*	Dogs, Ruminants	*mecA*, *aadD*	Pyoderma, otitis, wound infections	Direct contact with pets or livestock	[72,93]

**Table 3:** Comparative overview of AMR detection tools in livestock and food systems.

Detection Method	Key Features	Strengths	Limitations	Application Settings	References
Disk Diffusion and Broth Microdilution (AST)	Standardized phenotypic testing (CLSI/EUCAST)	Cost-effective, globally accepted, easy to perform	Time-consuming; requires viable isolates	Farm, clinical, food labs	[2,8]
PCR/qPCR	DNA-based detection of resistance genes	High sensitivity and specificity; fast turnaround	Requires skilled personnel and equipment	Farm, food labs, veterinary clinics	[164,165]
LAMP	Isothermal amplification without thermocycler	Rapid, field-deployable, visual readouts	Limited multiplexing; primer design sensitive	On-farm, LMICs, point-of-care	[168,169]
CRISPR-based Diagnostics (e.g., SHERLOCK/DETECTR)	Cas enzyme-guided nucleic acid detection	Ultra-sensitive; portable formats emerging	Not yet widely validated/commercialized	Research, prototype diagnostics	[170,177]
WGS	Comprehensive resistome and MGE detection	Source tracking; high resolution	High cost, infrastructure intensive	Reference labs, outbreak response	[38,39]
Shotgun Metagenomics	Culture-independent resistome profiling	Captures uncultivable/rare taxa; ARG landscape	Expensive; complex bioinformatics	Environmental, food, manure, wastewater	[14,35]
MALDI-TOF MS (±functional resistance assays)	Protein spectral ID; adjunct resistance workflows	Rapid, cost-effective ID; expanding AMR applications	Limited to known databases; phenotypic confirmation needed	Clinical and food microbiology labs	[186,187]
Optical/Imaging-based Rapid AST (e.g., DL-enhanced microscopy, Raman/SERS)	Growth/metabolic readouts within hours	Rapid phenotypic detection; high categorical agreement	Expensive instrumentation; early-stage validation	Clinical research, pilot veterinary settings	[160,162]

**Table 4:** Public health risks and One Health barriers linked to AMR in livestock.

Risk Domain	Key Findings	References
Foodborne Illness	Resistance detected in *Salmonella*, *Campylobacter*, and *E. coli* along the food chain; however, the quantitative human health burden attributable specifically to foodborne AMR remains poorly resolved.	[208,209]
Human Colonization	High LA-MRSA ST398 colonization among livestock workers; environmental reservoirs (e.g., barn dust) sustain transmission pressure.	[210,211]
Occupational Exposure	Farmers, veterinarians, transporters, and slaughterhouse workers show increased carriage and occasional infections with LA-MRSA, ESBL-producing Enterobacterales, and fluoroquinolone-resistant *Campylobacter*.	[212]
Hospital Spillover	Rising clinical infections with LA-MRSA CC398 (including bloodstream infections) closely related to porcine isolates in Europe.	[59,205]
One Health Barriers in LMICs	Fragmented governance and budgets, limited laboratory and genomic capacity, poor cross-sector data interoperability, and underregulated veterinary antimicrobial access/use.	[19]
Stewardship Needs	Tiered diagnostics (LAMP/qPCR to WGS/metagenomics), interoperable AMR/AMU data systems, harmonized CIA restriction lists, veterinary prescription auditing, farm-level alternatives (vaccines, probiotics, phages, precision husbandry), and sustained, multi-ministry financing with joint accountability indicators.	[99]

**Table 5:** Mitigation strategies to curb AMR in livestock production. A concise summary of actionable pillars, exemplar interventions, evidence of impact, and key enablers/requirements for implementation, with particular attention to scalability in LMICs.

Pillar/Domain	Specific Interventions	Evidence/Outcome	Reference(s)
Biological alternatives	Vaccines (e.g., *Salmonella*, *E. coli*, *Pasteurella*)	Reduced disease incidence and AMU; supports removal of antibiotic growth promoters (AGPs).	[213]
Farm-level AMS and biosecurity	Enhanced hygiene and biosecurity; targeted diagnostics; veterinary oversight; strict withdrawal periods	25–28% reduction in total AMU per pig in Danish farms; improved animal health outcomes	[23,217]
Precision agriculture and AI	Farm sensors; metagenomic surveillance; ML models for AMR prediction and targeted intervention	Early detection of resistance hotspots; real-time stewardship guidance; data-enabled decision-making	[215]
Policy and Regulation	EU AGP bans; Codex AMR risk frameworks; national AMU monitoring (e.g., VetStat in Denmark)	Regulatory enforcement paired with benchmarking tools; global adoption of best practice models	[218]
Case Study: Yellow Card	Benchmarking AMU per farm; thresholds for intervention; veterinary audits when limits exceeded	Demonstrated effectiveness in reducing AMU sustainably; scalable model for AMR control	[23,216,219]

## Data Availability

Not applicable. No new data were created or analyzed in this study.
